# 301. Impact of Prescribing Ertapenem Thrice Weekly Over Daily In Patients on Hemodialysis

**DOI:** 10.1093/ofid/ofaf695.103

**Published:** 2026-01-11

**Authors:** Surafel MULUGETA, Suzette Gendjar, Vince Procopio, Bhanujit Sabharwal

**Affiliations:** Henry Ford Health, Detroit, Michigan; Henry Ford Health System, Detroit, Michigan; Henry Ford Macomb Hospital, Detroit, Michigan; Henry Ford Hospital, bloomfield Hills, Michigan

## Abstract

**Background:**

Ertapenem (ETP) is an intravenous (IV) carbapenem used to treat resistant Gram-negative infections. In hemodialysis (HD) patients, ETP is dosed 500 mg daily. Emerging but limited data suggests an alternative post-HD dosing of 1 gram thrice weekly (TIW) may be safer, promotes vein-preservation strategy, and is more convenient for patients on outpatient parenteral antimicrobial therapy (OPAT). This study compares the outcomes of HD patients treated with ETP 500 mg daily versus 1 gram TIW.

**Figure:**
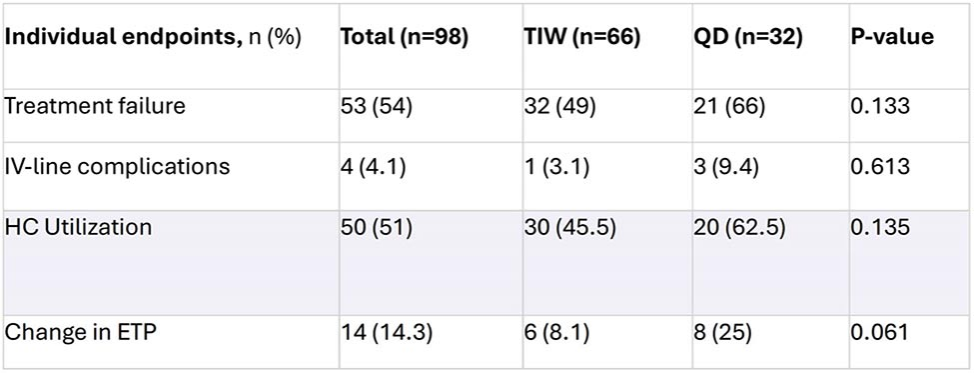
Individual Components of the Primary Composite Endpoint (Treatment Failure) Abbreviations; PICC: Peripherally inserted central catheter, HC: Healthcare, ETP: Ertapenem, IV: Intravenous, QD: Daily Definitions; IV-lines: Peripherally inserted central catheter or midline, HC: Emergency department or urgent care or all-cause readmission

**Figure:**
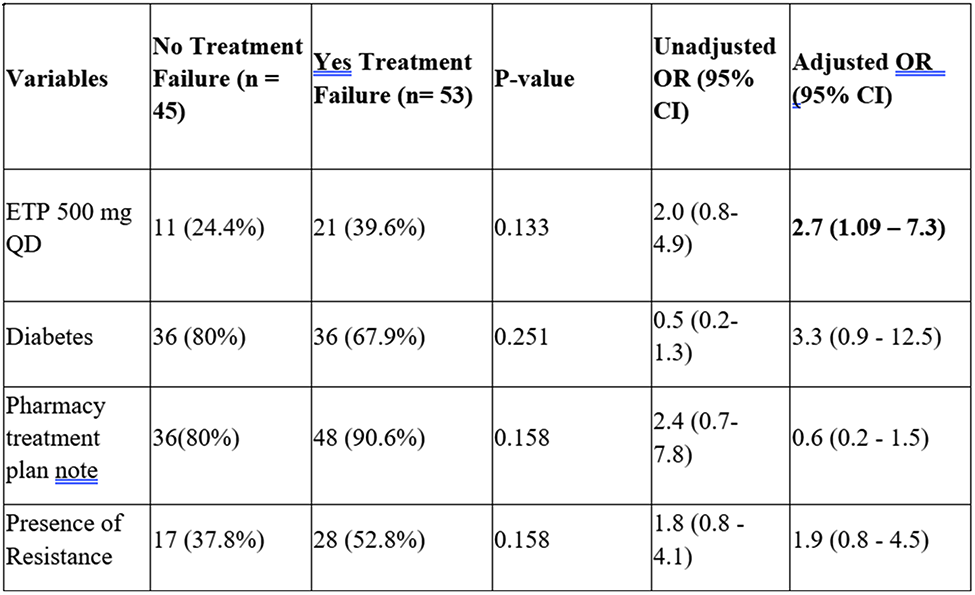
Logistic Regression Analysis of Characteristics Associated with Treatment Failure Abbreviations; Ertapenem, QD: Daily, OR: Odds Ratio, CI: Confidence Interval

**Methods:**

Institutional Review Board (IRB) approved, retrospective cohort study of hospitalized adult HD patients discharged on ETP from 7/1/2019 - 10/1/2024. Primary endpoint was treatment failure (TF), defined as IV-line complications, change in ETP therapy, or healthcare utilization, all within 60 days of being discharged. Secondary endpoints included efficacy, safety, and transition-of-care variables such pharmacist infection-treatment plan note completion. Bivariate analysis and logistic regression were implemented.

**Results:**

98 patients were included: 66 in TIW and 32 in daily group. Baseline characteristics were similar between groups, with the majority being older African American males (59%) with diabetes (74%) who received ETP for osteomyelitis (31%). Duration of therapy and hospitalization were similar. Compared to daily group, the TIW group experienced 72% fewer IV-line placement (p< 0.001) and a higher number of completed pharmacy treatment plan notes (92.4% vs. 71.9%, p=0.012). TF (Table 1) as well as rates of readmission, mortality, and adverse events were similar. After adjusting for confounders, ETP 500 mg daily regimen was associated with an increased odds of experiencing TF (Table 2).

**Conclusion:**

In HD patients requiring ETP, the alternative dosing regimen of 1 gm TIW is a patient and OPAT -friendly dosing regimen that promotes vein preservation strategy without sacrificing efficacy or safety.

**Disclosures:**

All Authors: No reported disclosures

